# Developing the next generation of renewable energy technologies: an overview of low-TRL EU-funded research projects

**DOI:** 10.12688/openreseurope.15276.2

**Published:** 2023-12-28

**Authors:** Laura María Pérez Caballero, Fernanda Neira D'Angelo, Roman Tschentscher, Axel Gottschalk, Ahmed M. Salem, Daniel Carbonell, Mihaela Dudita-Kauffeld, Arnaud Bruch, Eleonora Alamaro, Luca Pasquini, Paola Ceroni, Anastasia Grozdanova, Stefania Privitera, Bart Vermang, Philip Schulz, Davide Mencarelli, Luca Pierantoni, Michele Midrio, William Leithead, Ignacio Gurruchaga, Robert Haberl, Jasper Vermaut, Michael Kauffeld

**Affiliations:** 1 Innovation Area, R2M Solution Spain S.L., Madrid, 28032, Spain; 2 Chemical Engineering and Chemistry, Eindhoven University of Technology, Eindhoven, 5612 AZ, The Netherlands; 3 Process Chemistry and Functional Materials, Sintef Industry, Oslo, 0314, Norway; 4 Institute of Process Engineering, Bremerhaven University of Applied Sciences, Bremerhaven, 27568, Germany; 5 Department of Energy, Systems, Territory and Constructions Engineering (D.E.S.T.E.C.), University of Pisa, Pisa, 56122, Italy; 6 Mechanical Power Department, Faculty of Engineering, University of Tanta, Tanta, 31521, Egypt; 7 SPF Institute for Solar Technology, OST Eastern Switzerland University of Applied Sciences, Rapperswil, 8640, Switzerland; 8 Atomic Energy and Alternative Energies Commission, France, Grenoble, 38054, France; 9 AMIRES, Prague, 162 00, Czech Republic; 10 Department of Physics and Astronomy, University of Bologna, Bologna, 40126, Italy; 11 Department of Chemistry "Giacomo Ciamician", University of Bologna, Bologna, 40126, Italy; 12 Institute for Microelectronic and Microsystems (IMM), National Research Council, Italy, Catania, 95121, Italy; 13 imo-imomec, Hasselt University, Hasselt, 3500, Belgium; 14 imo-imomec, Imec, Genk, 3600, Belgium; 15 imo-imec, EnergyVille, Genk, 3600, Belgium; 16 Institut Photovoltaïque d’Île de France (IPVF), CNRS, Palaiseau, 91120, France; 17 Marche Polytechnic University, Ancona, 60121, Italy; 18 University of Udine, Udine, 33100, Italy; 19 Wind Energy and Control Centre (WECC), University of Strathclyde, Glasgow, G11XQ, UK; 20 Federation of European Heating, Ventilation and Air Conditioning Associations, Brussels, 1050, Belgium; 21 Institute of Refrigeration, Air Conditioning and Environmental Engineering, University of Applied Sciences Karlsruhe, Karlsruhe, 298R+86, Germany

**Keywords:** renewable energy, innovative technologies, research and development, net-zero greenhouse gas emissions

## Abstract

A cluster of eleven research and innovation projects, funded under the same call of the EU’s H2020 programme, are developing breakthrough and game-changing renewable energy technologies that will form the backbone of the energy system by 2030 and 2050 are, at present, at an early stage of development. These projects have joined forces at a collaborative workshop, entitled ‘ *Low-TRL Renewable Energy Technologies*’, at the 10th Sustainable Places Conference (SP2022), to share their insights, present their projects’ progress and achievements to date, and expose their approach for exploitation and market uptake of their solutions.

## Introduction

The growing importance of renewable energy to enable a faster transition to a net-zero greenhouse gas emissions EU economy by 2050 is increasing demand in advanced energy infrastructure. In 2018, the revised Renewable Energy Directive entered into force, as part of the ‘Clean energy for all Europeans’ package, establishing a binding Renewable Energy Sources (RES) target for 2030 of at least 32% of the overall consumed energy in the EU ^1^. However, the renewable energy technologies that will form the backbone of the energy system by 2030 and 2050 are still at an early stage of research and development. Bringing new energy conversion solutions, novel renewable energy concepts and innovative renewable energy uses faster to the commercialisation stage, taking into account social acceptance as well as the need for secure and affordable energy supply, is challenging. New technologies should not only have a strong commercial potential but also a lower environmental impact and lower carbon emissions, higher resource and energy conversion efficiency, as well as higher availability for different market and operating environments than the currently existing renewable energy technologies. In this line, a number of research and innovation (R&I) projects, funded by the European Union’s Horizon 2020 research and innovation programme, are developing breakthrough and game-changing solutions to tackle the above challenges and strengthen the EU’s global leadership in technologies for the exploitation of renewable energy sources.

A group of eleven European projects ( *i.e.*, HIGFLY, EBIO, FLEXI-GREEN FUELS, REGEN-BY-2, TRI-HP, ComBioTES, CONDOR, TELEGRAM, PERCISTAND, GreEnergy, and X-ROTOR), funded under the H2020-LC-SC3–2018–2019–2020 call ( *Building a Low-Carbon, Climate Resilient Future: Secure, Clean and Efficient Energy*), joined forces on a clustering workshop at the Sustainable Places 2022 (SP2022) Conference ^2^. This workshop, entitled ‘ *Low-TRL Renewable Energy Technologies*’, brought together projects dealing with the challenge of researching, developing and demonstrating the technological feasibility of innovative, efficient, flexible, cost-competitive, low-carbon-emission, environmentally safe and socially acceptable technologies, which are still at a low Technological Readiness Level (TRL 3-4). The representatives of the participating projects had the opportunity to share their insights, present their projects’ progress and achievements to date and expose their approach for exploitation and market uptake of their solutions, followed by an interactive discussion to exchange ideas, knowledge and best practices, find potential synergies in the R&I work being developed, and identify possible cooperation on cross-cutting issues to maximise their combined impact. A summary of the work presented during the workshop by each of these projects can be found in the following sections.

## HiGee to furanic-based jet-fuel technology, the HIGFLY project

The main aim of the HIGFLY project ^3^ is to develop sustainable aviation fuels from abundant second-generation feedstock in order to mitigate the environmental impact of aviation. HIGFLY plans to achieve this by targeting sustainable feeds, maximising resource efficiency, minimising energy demands, increasing process scalability and minimising environmental impact, while also decreasing the cost and accelerating the uptake of bio-based sustainable aviation fuels, increasing their share in the EU market.

Specifically, the HIGFLY project will develop and demonstrate, at TRL3–4, novel reactor and separation technologies and a robust and continuous catalytic process to produce and purify jet fuel precursors from C5 biorefinery streams with 80–90% carbon efficiency; and will also evaluate catalytic routes to valorise light oxygenates present in aqueous side-streams to produce hydrocarbons and hydrogen. The suitability of HIGFLY’s sustainable aviation fuel will be assessed along with the sustainability, environmental and social impacts of the production process by evaluating abundant and sustainable feedstocks, and conducting techno-economic and life cycle assessments (of the entire value chain, from feedstock to bio jet fuel).

The main achievements of the HIGFLY project to date are the following:

a) 35 new catalyst materials have been synthesised, characterised and tested, with two being identified as highly attractive.
b) New, sustainable solvents have been discovered via AI-based modelling using quantum mechanical data, with an estimated 5–10 times greater performance than conventional solvents.
c) Catalysts have been formed into pellets and extrudates for testing in the HiGee reactor and have shown to be both scalable and highly efficient.
d) New reactors based on HiGee technology that show enormous potential for process intensification due to higher mass and heat transfer rates over other conventional reactors. Results from experiments and modelling show a potential 50-fold decrease in reactor size with an increased yield of up to 90% compared to the technology currently available; and a significant reduction in the amount of humins, which allows for continuous operation and, therefore, a reduction in capital expenditures to produce furfural.

## Biofuels through electrochemical transformation of intermediate bio-liquids, the EBIO project

The EBIO project ^4^ develops sustainable routes for upgrading industrial bio-liquids in order to simplify storage, transport and further processing to fuels and platform chemicals. Specifically, EBIO targets the conversion of unstable compounds into stable intermediates, the removal of acids and the depolymerisation of high molecular weight fractions. EBIO’s innovative concept focuses on electrochemical upgrading of two typical industrially-available biomass-based liquefied feedstocks (i.e., black liquor and fast pyrolysis oil) through successive hydrogenation and decarboxylation. Process design and optimisation includes electrode materials, reaction cells, separation/purification, upscaling and integration into existing pulp mills and pyrolysis processes. Products targeted specifically will have a higher energy density and stability, lower averaged molecular weights, and less diverse oxygen functionalities in the molecules, as compared to the original feeds. This allows for better blending/mixing with existing refinery streams and results in higher overall yields (in terms of carbon in the product, such as chemicals and biofuels).

EBIO’s key expected achievement is the near-seamless integration of electrochemistry into biorefinery processes by combination of all the data using data treatment tools for flow sheeting, design, and impact analyses (yields and reaction rate/kinetics, energy balance, process efficiency, empirical links between the investigated process parameters/descriptors). Based on this, a detailed techno-economic evaluation is to provide a realistic estimation of the manufacturing cost (substrates availability and supply chain, future end-users and economic sustainability of the process). Further societal and environmental challenges and impacts are assessed, including barriers for social acceptance, impact of induced transport, as well as potential benefits (innovation potential, value added, employment) for regional economies, etc.

During the initial year of the project, efforts concentrated on: 1) lab scale screening and electrochemical process optimization and 2) preparative work for sustainability assessment studies. Process design studies have focused on setting up initial process flow sheets for electrochemical conversion processes integrated into the bio-liquid production process. In addition, design requirements to minimise changes and investment costs were established, which guide the experimental work packages. Summarising EBIO’s progress so far, electrochemical treatment of the black liquor is technically feasible. Electrochemical pyrolysis liquid conversion is preferably performed after an initial fractionation process separating oil and aqueous phase. The experimental studies focused on identification of suitable electrode materials as well as the establishment of the operational window. This lays the groundwork for paired electrochemical tests combining anodic oxidation and cathodic reduction processes. Separation of oxidised intermediates of higher value is technically feasible, for example by adsorption or extraction. For fuels production most feasible seems the consecutive electrochemical or catalytic reduction. Lab-scale cell prototypes were designed and provided to project partners as a basis for bench-scale continuous tests. Preliminary results of societal impacts consist of pre-selection of a set of impact categories, criteria and indicators for the analysis. This has been developed in dialogue with project partners and will be used as basis for further dialogue with the other project partners and external stakeholders, to come up with a final set of factors.

During the ' *Low-TRL RES technologies*' workshop at the SP2022 Conference, the goals, objectives and current status of the EBIO project was presented. Synergies with complementary projects focusing on biofuels projects have been identified with respect to feedstock provision, analysis of complex bio liquids and scale up. Possibilities for joint research but also communication and dissemination activities have been identified.

## Flexible and resilient integrated biofuel processes for competitive production of green renewable jet and shipping fuels, the FLEXI-GREEN FUELS project

The FLEXI-GREEN FUELS project ^5^ is a research and innovation action, with duration of 3 years (2021–2023), on which 13 beneficiaries joined forces to develop innovative technologies to convert biomass residues and wastes like forest residues and the organic fraction of municipal solid waste into fuels for aviation and marine fuels.

Lignocellulosic biomass is fractionated into lignin, hemicellulose and cellulose fractions. Cellulose is pretreated by enzymatic hydrolysis in order to obtain sugars. As lipids are far superior bio-crudes towards hydrocarbon fuels, the FLEXI-GREEN FUELS project develops and optimises three efficient methods to convert sugars to lipids, namely: 1) fungal fermentation, 2) algae dark fermentation, and 3) lipid rich larva production. Additional catalytical and thermochemical conversion technologies are also under investigation to convert other intermediates into crude biofuels. Bio-hydrogen from microbial electrolysis is used for hydrotreatment of the crude biofuel to turn it into high-quality fuels for aviation. Fuel tests are included for bunker-like shipping fuels and jet-like aviation fuels. Finally, a large-scale marine engine test is scheduled towards the end of the project. All activities are complemented by modelling and simulation activities, techno-economic evaluation as well as life cycle impact assessments. The advantages of the new biofuels over conventional aviation and shipping fuels are quantified by benchmarking.

The coordinator of the collaborative FLEXI-GREEN FUELS project, Prof. Dr.-Ing. Axel Gottschalk (Bremerhaven University of Applied Sciences), is convinced that liquid fuels will remain dominant in future aviation and marine applications. Both green alternative synthetic fuels as well as green bio-based fuels will be required to meet the demands for global mobility and trading in a sustainable way.

## Next Renewable multi-generation technology enabled by two-phase fluids machines, the REGEN-BY-2 project

The REGEN-BY-2 project ^6^, launched in September 2020, is aimed at developing a first-of-its-kind lab-scale prototype of a flexible, highly efficient thermodynamic cycle for Combined Heating and Power (CHP), Combined Cooling and Power (CCP) and Combined Cooling Heating and Power (CCHP), driven by renewable thermal energy sources ( *i.e.*, biomass, geothermal, solar, waste heat recovery). The key of REGEN-BY-2’s flexible and efficient tri-generation system is the design, construction and testing of two-phase fluid machines (scroll expanders and compressors), capable of operating in the bi-phasic region of working fluids ( *i.e.*, biphasic fluids, composed of both liquid and vapour phases). The patented thermodynamic cycle is constituted by a proper combination of Carnot cycles at different temperature levels. The project targets to select the most appropriate working fluid for achieving higher flexibility than other tri-generation technologies. The approach consists of, firstly, designing the prototypes and simulating the process via mathematical models and Computational Fluid Dynamics (CFD) analysis, as well as performing a thermodynamic sensitivity analysis. Secondly, the lab-scale prototypes will be built during the second half of the project, through the design technical specifications. Lastly, REGEN-BY-2 will perform experimental tests (using a biomass boiler as heat source for the system operation) for the validation of the lab-scale prototypes of the expander and the compressor, as well as the whole tri-generation plant, including the control system.

To date, the design as well as the dynamic and off-design modelling of the different components of the REGEN-BY-2 technology have been demonstrated. The selection of different components has been processed, and the build-up of the lab-scale prototype of the tri-generation system will start soon. Although the project aims to position the technology at TRL4, there are plans to bring the solution to TRL9 (complete marketability) by 2030. These plans include a research and development roadmap towards higher TRLs, as well as a commercialisation roadmap including regulatory and standardisation issues, aimed at identifying and reducing the potential market and social barriers. The economic performance of the REGEN-BY-2 technology in the future RES-based EU energy system will be assessed. Also, the replicability of REGEN-BY-2 in different countries will be evaluated, considering local potential grid impact and presence of feed-in tariffs for renewable power plants.

## Trigeneration systems based on heat pumps with natural refrigerants and multiple renewable sources, the TRI-HP project

Joining forces for a clean energy transition, the partners from EU-funded TRI-HP project ^7^ have a holistic approach for providing energy to multi-family residential buildings using heat pumps (HPs) based on natural refrigerants. TRI-HP stands for trigeneration heat pump systems that provide heating, cooling and electricity with an on-site renewable share of 80 % reducing the installation costs by 10–15 %. Stakeholders' needs, building demand characteristics, local regulations and social barriers, are considered to be able to provide the most appropriate technical solutions.

Current commercial heat pump units often rely on highly polluting synthetic F-gases as refrigerants. These are either HFCs with high global warming potential (GWP) or HFOs with low GWP. However, HFOs lead to the formation of trifluoroacetic acid (TFA) as atmospheric breakdown product. HFCs are considered in the EU’s F-gas regulation, while HFOs are expected to be regulated under the REACH regulation. Regardless of the various regulations, the precautionary principle should justify the use of natural refrigerants like propane or carbon dioxide.

**Figure 1.**
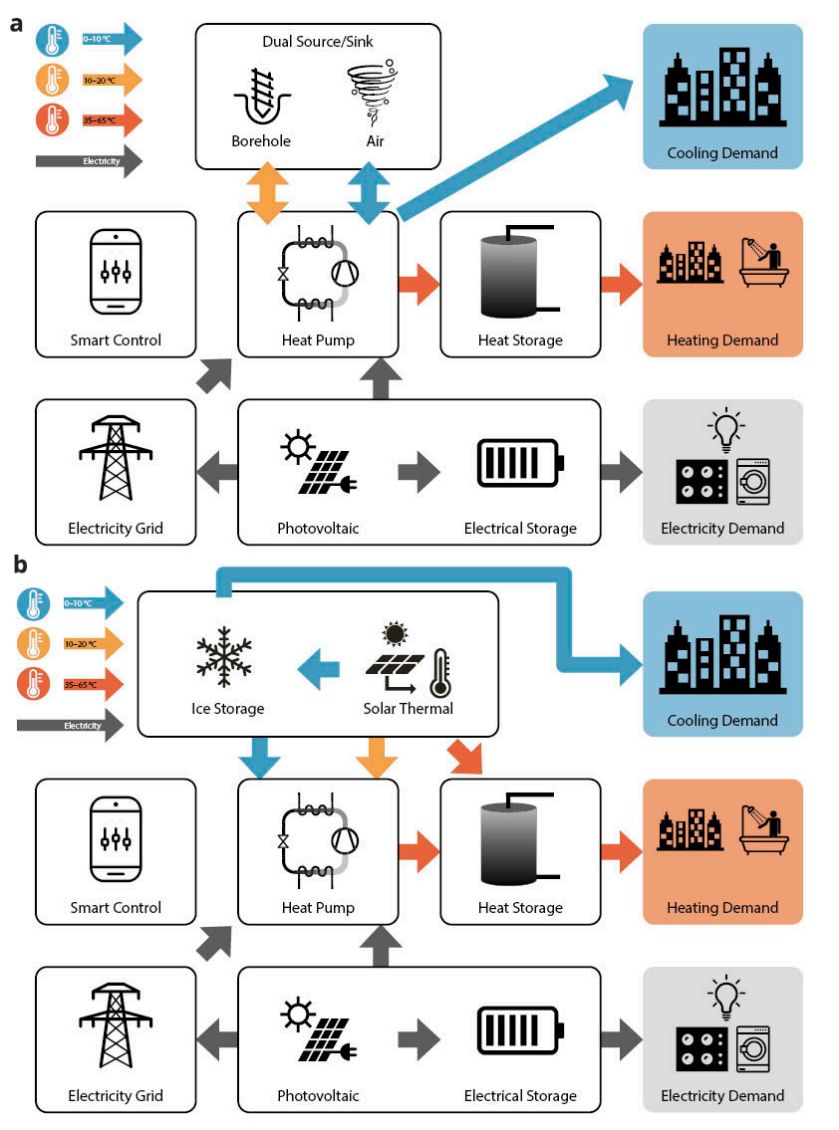
TRI-HP system concept with dual ground/air source ( **a**) and with solar and ice-slurry ( **b**) as intermediate storage.

The TRI-HP systems are based on electrically driven natural refrigerant heat pumps coupled with renewable electricity generators (PV), and are using cold (ice slurry) and warm thermal as well as electrical energy storages. They provide heating, cooling and electricity to multi-family residential buildings with a self-consumed renewable share of 80%. The flexibility of TRI-HP systems is achieved by allowing three heat sources: solar (with ice/water as intermediate storage medium), ground and ambient air. Two system concepts are developed for two different combinations of heat sources (see Figure 1):

i) Dual-source system that exploits both air and ground as heat sources and sinks using a reversible concept featuring a condenser-evaporator unit which uses air, brine or both. This system allows to reduce the length of boreholes by 50% for the same efficiency as regular ground source heat pumps.
ii) Solar ice-slurry system which uses solar thermal energy as heat source with ice slurry as intermediate storage during periods with insufficient sun. In moderate climates like central Europe, it can also provide free cooling. Using ice-slurry instead of conventional ice storage can decrease installation costs by 10%.

The development of the heat pump unit itself and the whole system solution was done hand-in-hand with simulations and measurements. The first prototype has been tested in static conditions under a wide range of boundary conditions. The insights of those measurements were then used to improve the system. For example, the COP of the propane-ice HP has improved by 11% between the first and the second test campaign, in the case of the CO _2_ HP a 14% higher heating capacity and a 18% higher COP was achieved. Moreover, the interaction of the whole system and its hydraulics with control strategy and the user behaviour was shown in dynamic real-life operation. Thereby, the suitable integration based on the requirements of the refrigerant cycle was shown as well as a stable and efficient operation with sub-0 temperatures on the source side. TRI-HP innovations reduce the system installation cost by at least 10–15% compared to current heat pump technologies with equivalent energetic performances. Moreover, TRI-HP systems are supported by an advanced management control which makes use of a model predictive control to manage electricity, heat and cold through real-time optimisation of the energy flows based on dynamic price signals. This control has allowed to reduce systems operational costs by at least 10%. The implementation of TRI-HP systems will reduce GHG emissions by 75% compared to gas boilers and air chillers.

## Compact bio-based thermal energy storage for buildings, the ComBioTES project

The increasing ratio of inherently intermittent renewable energy sources into the global electric mix makes energy storage as one of the crucial aspects of the ongoing transition towards sustainable energy production. The evolution towards higher renewable energy generation leads to higher fluctuations in the grid outputs, making the energy storage necessary to smooth these fluctuations and to reduce the demand peak. At residential scale, thermal end-uses ( *i.e.*, space heating, hot tap water preparation, cooling) represent one of the biggest consumers of electricity in European countries, and are also one of the causes of electricity consumption peaks during the day. This is the context that brought ComBioTES to life. ComBioTES ^8^ is an H2020 research and innovation project, which started in November 2019 and has a duration of 48 months.

**Figure 2.**
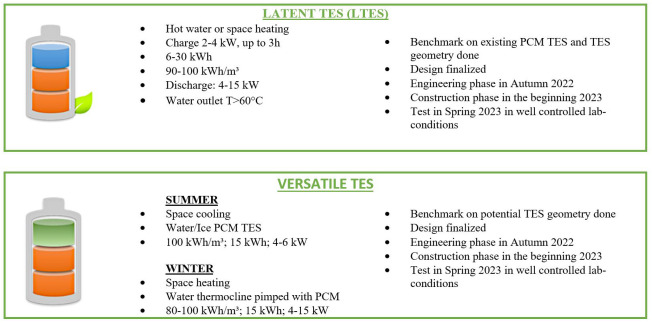
ComBioTES PCM TES and Versatile TES characteristics.

ComBioTES develops a modular solution based on two thermal energy storages (TES, see Figure 2) for heating, hot tap water and cooling, especially addressed to thermal end-uses in buildings, which will be fully adapted for electricity load shifting. A PCM (Phase Change Material) TES and a “versatile” TES are currently under design and will be built in 2023, with their first characterization also in 2023. The PCM TES uses the enthalpy of melting/solidification of a bio-based material to store/release the energy, for hot tap water and space heating applications. The “versatile” TES is an innovative TES used as a classical water thermocline in winter to produce space heating and converted into water/ice storage during summer to allow space cooling.

Both storages are planned to be manufactured in the beginning of 2023, with experimental characterization in well controlled laboratory conditions during Spring/Summer 2023. This step at lab-scale will be followed by a 1 year-long test phase in realistic-to-real operating conditions in the sites and applications shown in Table 1.

**Table 1.** ComBioTES demo-sites description.

SITE	DESCRIPTION	APPLICATION
DTU (Denmark Realistic conditions CEA (France) Test platform	Representative house Fully instrumented	Space heating Hot tap water Heat pump pre-heating
IEO (Poland) Real operation	200 m ^2^ single-family house Natural gas + heat pump + PV	Hot tap water
IEO (Poland) Real operation	240 m ^2^ office building, 25 people Gas heater + solar thermal	Space heating

ComBioTES is aiming at positioning its solution in the fast-growing energy storage market, offering a competitive product endowed with a user-friendly control system and able to ensure significant savings on domestic electricity bills. In fact, the project will finish in 2025 and in the upcoming 3 years it is expected that the two industrial partners will invest in the solution and include ComBioTES in their portfolio.

## Combined sun-driven oxidation and CO _2_ reduction for renewable energy storage, the CONDOR project

The CONDOR project ^9^ is targeted at building up a modular laboratory demonstrator for solar driven production of energy carriers ( *i.e.*, methanol and dimethyl ether) and added value chemicals from biomass valorisation. CONDOR is aimed at the production of fuels by using carbon dioxide (CO _2_) as feedstock and sunlight as the sole energy source. The project proposes a photosynthetic device made of two compartments:

● Compartment 1: photoelectrochemical cell that splits H _2_O (water) and CO _2_ (carbon dioxide) and generates oxygen and syngas, a mixture of H _2_ (hydrogen) and CO (carbon monoxide);

● Compartment 2: (photo)reactor that converts syngas (produced in compartment 1) into fuel CH _3_OH (methanol) or dimethyl ether (DME) via bi-functional heterogeneous catalysts.

The final target is a full photosynthetic device – a laboratory-scale prototype that couples Compartment 1 and Compartment 2 – capable of a 3-month continuous outdoor operation with a solar-to-DME conversion efficiency of 4.5% (O _2_ or Cl _2_ evolution) or 6% (biomass oxidation). In terms of the work plan, the two compartments will be developed in parallel in the first 36 months. Compartment 1 is developed through synthesis and modelling of molecular catalysts, preparation of photoanodes for water or biomass oxidation, and (photo)cathodes for syngas production, and finally assembled and tested. For Compartment 2, which is aimed at syngas conversion to fuels by thermal or photoactivated processes, synthesis of different proximity catalysts and evaluation of their performance, in terms of activity and selectivity, is performed. In addition, CONDOR has activities focused on the characterisation of the molecules and nanomaterials used in both compartments from a structural, photophysical and electrochemical point of view. The testing and validation of the complete CONDOR prototype are planned to start in the second half of the third year of the project.

CONDOR aims at increasing the overall TRL of the system from 2–3 to 4. The full system will be demonstrated in outdoor settings (TRL4) at CNR-ISOF in Bologna (Italy). The project is expected to implement a laboratory-scale prototype comprising both compartments and to test it over a 3-month outdoor operation. The final prototype will be finalised and tested at the beginning of 2025. Beyond the end of the project, further scale-up is expected to occur. HyGear is CONDOR’s system integrator and the partner appointed to bring it to the market. The targeted customers will be chemical and petrochemical industry, oil and gas companies as well as ‘smart’ cities. South Europe is currently considered as the most suitable location for the first CONDOR system instalment, as a place combining advanced industry with a high solar irradiance index.

## Toward an efficient electrochemical green ammonia cycle, the TELEGRAM project

Ammonia is one of the most important chemicals used as fertiliser and in many other applications. Its production is presently achieved through a process responsible for about 1–2% of total CO _2_ emissions worldwide. Ammonia is also a potentially formidable energy vector, with high energy volumetric density, large hydrogen content and, unlike H _2_, it can be liquefied at room temperature and low pressure for storage and transportation. It could be therefore adopted to mitigate the intrinsic fluctuations of renewable energy sources. The main goal of the TELEGRAM project ^10^ is to demonstrate, at a laboratory scale, a complete green ammonia carbon energy cycle which, will produce ammonia by electrochemical synthesis in a reactor powered by RES, and will use the produced ammonia to get back electricity in a direct ammonia fuel cell.

In order to achieve the desired objective it is crucial to develop the two key enabling electrochemical devices and to select proper catalysts. The TELEGRAM Consortium is focusing on three different approaches: 1) novel high entropy alloys (HEAs) and 2) nanostructured catalysts for the ammonia synthesis, and 3) bi-metallic catalysts using earth abundant materials for ammonia oxidation. For the high entropy alloys, the compositions with better estimated activity have been selected through atomistic simulations among 3000 alloys. The more promising have been produced and characterised. The best ammonia production rate (1.6×10 ^−10^ cm ^−2^·s ^−1^) has been experimentally achieved with a CoCrFeMnNi alloy, with a faraday efficiency of 20%. Gold based (Au loading <0.02 mg·cm ^−2^) and iron-oxide based nanostructured catalysts have been prepared and characterised for ammonia synthesis. A remarkable faraday efficiency of 40% has been achieved with Mo-Au nanoparticles. Bimetallic catalysts (NiCu) have been prepared by electrodeposition on stainless steel felt and tested. The first results are promising, currently comparable with Pt-based catalysts, although the elimination of the noble metals is paid in terms of less stability and higher overvoltage.

The possibility of producing green ammonia, with zero CO _2_ emissions, using earth-abundant materials, as proposed in the TELEGRAM project, is of great interest not only for the use of ammonia as energy vector, but also because onsite distributed production of green ammonia would contribute to the decarbonisation of the global economy of the future, supplanting the energy intensive process and reducing the transportations. The expected increased access to ammonia as fertiliser is also associated with social and economic benefits, especially for developing countries or isolated regions with lack of infrastructures. The technologies TELEGRAM is addressing, however, are still at a nascent stage, with TRL around 3. Therefore, there is a strong impact related to technical and innovation aspects. The basic knowledge the TELEGRAM Consortium is acquiring is fundamental to collect scientific proofs of the technological feasibility of its basic concept and to build a know-how crucial for designing better performing devices, assessing innovative strategies to produce green fuels.

## Development of all thin-film perovskite on CIS tandem photovoltaics, the PERCISTAND project

The premise of the PERCISTAND project ^11^ is the search for efficient and sustainable ways to generate electric power. This endeavour constitutes a major building block to addressing climate change and transforming our energy economy. Today, solar photovoltaic (PV) modules ( *i.e.*, the direct conversion of sunlight into electricity) stand out in the electric power sector with the steepest acceleration in growth rates of cumulative installed capacity, which has been projected to exceed 1 TW as of 2022 ^12^.

Tandem photovoltaic devices based on CIGS and perovskite solar cell (PSC) sub-cells offer the implementation of all-thin-film tandems with all the advantages of thin-film technology, together with the possibility to achieve high efficiencies above the thermodynamic limit of single-junction solar cells. More precisely, the CIGS/perovskite tandem solar cell technology allows for low cost, lightweight, large-area roll-to-roll fabrication and lower integration and installation costs, finally promising lower energy costs compared to current technologies. To this date, record power conversion efficiency (PCE) of slightly over 24% and 27% have been achieved for monolithic two-terminal (2T) and stacked four-terminal (4T) tandems, respectively. The crucial step for coupling CIGS and PSC devices in one tandem cell lies in the optimization of the layer stack with respect to light management and electric current collection, which include the growth of the transparent electrode layers. To ensure compatibility, PERCISTAND developed and implemented common layouts for both 0.5 cm ^2^ cells and 10 cm ^2^ mini-modules. The approach allows reporting the performances of actual, fully functional 4T cells, whose performance can be externally certified. The champion devices with a 0.5 cm² active area produced in the PERCISTAND project reached a PCE of 27.4%. Furthermore, 4T mini-modules were produced with efficiency as high as 17.5% and with low cell-to-module losses, demonstrating the efficacy of our patterning process.

The requirements mentioned above become even more stringent in monolithic 2T tandems, where cell performance requires current matching between top and bottom cells. This means that, in particular for the CIGS cell, it is necessary to tailor the photoactive band gap to make optimum use of the partial illumination in the spectral region above ~750 nm wavelength, which is transmitted through the perovskite cell stack. One means to realise a beneficial trade-off between gains in the open circuit voltage and losses due to reduced photoabsorption is by the design of band gap grading, *i.e.* Ga in-depth distribution in the CIGS absorber film. Hence, PERCISTAND’s approach to achieve high performance monolithic interconnected perovskite/CIGS 2T tandem solar cells rests on four major building blocks. i) The adaptation of the CI(G)S bottom cell including control over the surface roughness; ii) growth and optimization of the PSC layer stack on top, notably with focus on the hole transport layer; iii) the design of the tunnelling/recombination junction connecting the two sub-cell;, and iv) the implementation of light management structures for improved current matching. The combination of these improvements enabled the Consortium to establish record PCEs for 2T cells of 0.5 cm² size of 24.9% ^13^.

## Wideband optical antennae for use in energy harvesting applications, the GreEnergy project

Fossil fuel usage is continuously rising, which is causing global warming to accelerate; this trend will get worse in the next decades as the world's population rises and with increase of use of energy as a result of modernity. The main objective of the GreEnergy project ^14^ is to harness solar energy more effectively and affordably than current PV solar cell technology. The ground-breaking innovation of GreEnergy is to use the electromagnetic wave property of sunlight with a maximum efficiency of up to 44%, compared to 33% for the maximum efficiency of single junction PV cells. To this aim, nano-antenna arrays and nano-structured geometric diodes are investigated and tested in the project.

Clean energy still makes up a small portion of the total world energy consumption. Despite countless studies tackling this issue, none of them have made adequate progress. By using solar power more efficiently, the GreEnergy project aims to benefit humankind by saving money and energy. The system is set to be highly efficient, with an efficiency target of 20–40% of the overall system. The starting point is given by the design of the rectennas and the entire system, adhering to the GreEnergy project's system approach philosophy. Different system architectures, such as nano-antennas, nano-antenna arrays, and nano-diode structures, are being carefully explored. The GreEnergy system's definition has also been formalised, along with the system's components, technical standards, and modelling.

**Figure 3.**
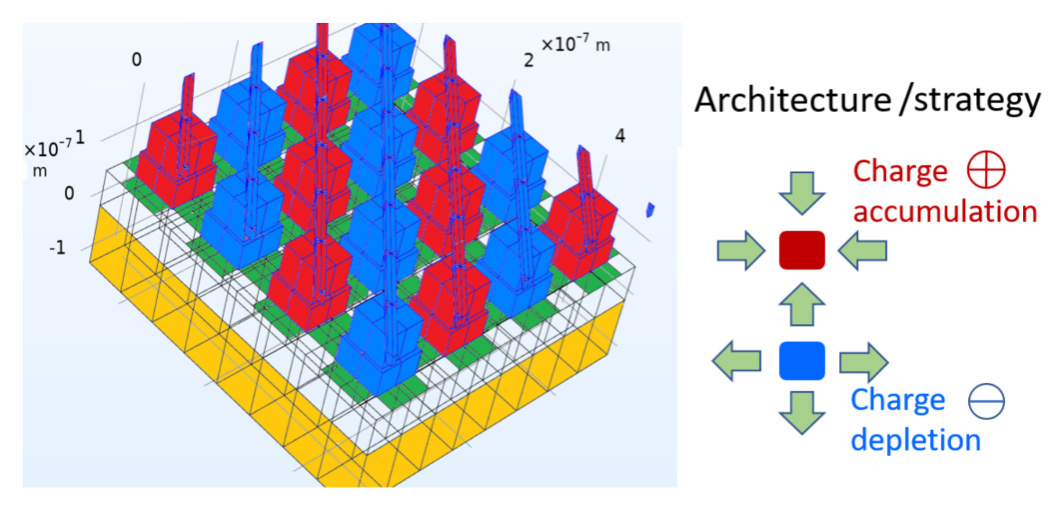
GreEnergy’s energy harvesting architecture.

Figure 3 shows the harvesting architecture proposed in the project: a metal-patch array acts as a receiving antenna, and nonlinear graphene elements (geometric diodes) provide rectification between cold and hot interconnected electrodes. Interconnected metal patches act as cold (blue) and hot (red) electrodes for sun light reception and rectification, by means of graphene-based geometric diodes (green areas). A bottom metal reflector (yellow) is separated from the patches by a dielectric layer.

The primary outcomes of the project so far are:

a) Initial low-frequency design of a graphene ballistic diode with close to 50% efficiency achieved through precise charge transport modelling;

b) Creation of an optical antenna construction model with 71% receiving efficiency, a record-breaking figure for solar light across a broad spectrum ^15^;

c) Creation and improvement of the manufacturing process for the optical antenna and diode;

d) Design, manufacture, and verification of various structural elements, such as electrode materials, electrolytes, separators, and current collectors for the energy storage device (supercapacitor or micro-supercapacitor); at the same time: evaluation of various interface circuitry configurations.

With the modelling and design of a very high efficiency antenna array (more than 70%) and ballistic diode (about 50%), which are above the state of the art, GreEnergy has already made enormous strides. At showing a prototype that achieves 20–40% efficiency for the integrated components, lab-scale technology validation at the system level is expected by the end of the project. Additionally, GreEnergy will benchmark the technology for upcoming commercialization and development. Consumers should receive a fair deal under the GreEnergy system, which should also promote the EU's and the global renewable energy leadership while speeding up the market penetration of clean energy. Promoting the use of green energy while reducing fossil fuel consumption will have a positive impact on the economy, human health and welfare, and reduce the threat of global warming.

## X-shaped Radical Offshore wind Turbine for Overall cost of energy Reduction, the X-ROTOR project

Wind power is now cost-competitive with conventional generation from fossil fuels. However, to maintain its rate growth through exploiting the wind resource in more inhospitable locations, such as far offshore in deep water, there is continuing pressure to reduce its cost of energy. The X-ROTOR project ^16^ seeks to address this challenge by substantially reducing the cost of energy from wind power through the development of a radical new wind turbine concept: the X-ROTOR concept. Given that the standard wind turbine concept, the horizontal axis wind turbine (HAWT), has been very successful and is at an advanced stage of development; to be successful, any new concept would need to achieve significant reductions in capital costs (CAPEX) and operation and maintenance costs (OPEX) costs. The X-ROTOR wind turbine concept was conceived to directly address these twin requirements.

**Figure 4.**
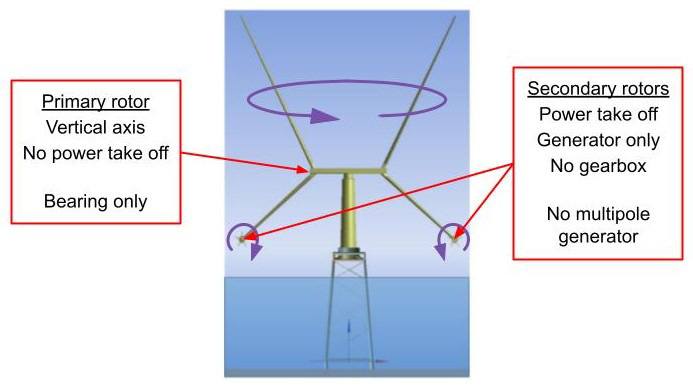
X-ROTOR wind turbine concept.

The X-ROTOR concept (see Figure 4) consists of a primary rotor that rotates around a vertical axis and secondary rotors that rotate about horizontal axes, the primary rotor’s sole purpose is to drive the secondary rotors attached to the ends of the lower blades; that is, unlike a conventional vertical axis wind turbine that, also has a rotor rotating about a vertical axis, it does not use it to drive a generator. The secondary rotors drive generators to deliver electrical power. Since it is induced by the rotation of the primary rotor, the wind speed experienced by the secondary rotors is very high. Consequently, the diameter of the secondary rotors is very small and their rotational speed very high. (On a 5 MW turbine with two secondary rotors, each would have a diameter of about 9.5m and a rotational speed of about 40 r/s.) There is, therefore, no need for a gearbox or multipole generator. The primary rotor aerodynamic torque is balanced by the combined secondary rotor thrust. The secondary rotors torque is balanced by the reaction torque from the generators.

With regard to CAPEX, the most significant reduction stems from the replacement of a conventional power-train by power take-off units consisting of a secondary rotor and a generator. This reduction is estimated in a previous feasibility study ^17^ to be roughly equivalent to a reduction in CAPEX of 5% for a three-stage DFIG, 10% for a three-stage PMG, 15% for a two-stage PMG and 32% for a DD PMG conventional 5 MW turbine. As the size of the turbine increases these reductions will become greater. With regard to OPEX, the X-ROTOR concept has several advantages. Major components such as secondary rotors and generators are at low height, about 25 m above sea level, and light, about 10 tonne, providing easy access with no requirement for heavy lift vessels. Power take-off is highly reliable since no gearbox or multipole generator is required. The secondary rotor and or the power take-off units could be detachable for transfer to shore for repair/maintenance. The turbine could continue to operate one power take-off unit non-operable. The resulting reduction in OPEX is estimated ^18^ to be roughly equivalent to a reduction in CAPEX of 53% for a three-stage DFIG, 49% for a three-stage PMG, 44% for a two-stage PMG and 24% for a DD PMG conventional 5 MW turbine. As the size of the turbine increases these reductions will become greater. The combined cost reduction from CAPEX and OPEX is potentially 20% to 25% for all four types of conventional turbines. Furthermore, the overall mass would be less than for a conventional HAWT.

The development of the X-ROTOR concept is at an early stage. The objectives for the X-ROTOR project are to take it from TRL1 to TRL3–4. State-of-the-art design tools, that currently exist for traditional offshore wind turbines, are not capable of modelling the unique characteristics of the X-ROTOR offshore wind turbine, consequently new design tools beyond the state-of-the-art are to be created. These advanced design tools include aeroelastic models, control models, structural models and maintenance models. By the end of the project the design tools will be used to deliver an exemplary design of the X-ROTOR concept and confirm the CAPEX and OPEX reductions previously identified.

## Conclusions

All the projects participating in the ‘ *Low-TRL Renewable Energy Technologies*’ workshop are developing innovative renewable energy technologies that aim to play a key role in building a sustainable energy system by 2030 and 2050, contributing to the decarbonisation of the EU economies. This includes, for example: new, more efficient, and cost-competitive energy generation and conversion technologies; innovative design tools for acceleration of wind energy technology development and increased life time extension; Sustainable fuels other than hydrogen for energy and transport application through ground-breaking conversion technologies; sustainable fuels for energy and transport application through ground-breaking conversion technologies; or novel very high efficiency thin-film photovoltaics concepts.

Each of the 11 projects presented in this article are addressing high-risk/high return technology developments for breakthrough renewable energy and fuel technologies, and their activities focus on providing knowledge and scientific proof of the feasibility of the proposed concepts, including their environmental, social and economic benefits. Although the innovative solutions that these projects are aiming to demonstrate (at TRL3-4) are different from one another, they will all face similar challenges in terms of bringing the technologies to TRL9 and exploiting them successfully in the market after the end of the R&I projects. For this reason, during the panel discussion at the SP2022 workshop, several questions were raised related to: i) the main barriers to innovation in energy generation systems and processes; ii) the foreseen challenges in bringing these new RES technologies to the market, and which measures are currently being taken in these R&I projects to overcome the identified challenges and ensure a successful commercialization in the future; and iii) the role that these low-TRL technologies will play in the future energy system of 2030 and 2050, whether they fit in a distributed generation scenario, and which need/gap in the market will they try to address/ fit into. These questions served for brainstorming ideas, knowledge exchange among the projects’ representatives (e.g., sharing their respective approaches for further R&D, technology roadmapping and/or exploitation plans) and debating different ways to address the common challenges. The workshop was a touchpoint, but the projects will continue the collaboration and discussions to ensure knowledge transfer, best practice exchanges and synergy identification to accelerate the energy transition and fulfill the ambitious objectives of the Clean Energy Package.

## Disclaimer

This publication reflects only the authors’ views, and the European Union is not liable for any use that may be made of the information contained therein.

## Figures and Tables

**Table 2.** Information for each H2020 Project contributing to this Open Letter.

Acronym	Project Title	Grant Agreement ID	Start date	End date	EU contribution
HIGFLY	HIGee to Furanic-based jet Fuel technologY	101006618	01/01/2021	31/12/2024	€ 3 994 322,50
EBIO	Biofuels through Electrochemical transformation of intermediate BIO-liquids	101006612	01/12/2020	30/11/2024	€ 3 988 603,75
FLEXI-GREEN-FUELS	Flexible and resilient integrated biofuel processes for competitive production of green renewable jet and shipping fuels	101007130	01/01/2021	31/12/2023	€ 3 993 006,25
REGEN-BY-2	Next REnewable multi- GENeration technology enabled by TWO-phase fluids machines	851541	01/09/2020	31/08/2024	€ 4 905 748,75
TRI-HP	Trigeneration systems based on heat pumps with natural refrigerants and multiple renewable sources	814888	01/03/2019	28/02/2023	€ 4 993 080,00
ComBioTES	Compact bio-based thermal energy storage for buildings	864496	01/11/2019	30/04/2025	€ 3 999 128,75
CONDOR	COmbined suN-Driven Oxidation and CO2 Reduction for renewable energy storage	101006839	01/11/2020	31/10/2024	€ 3 989 116,25
TELEGRAM	Toward efficient ELEctrochemical GReen AMmonia cycle	101006941	01/11/2020	30/04/2024	€ 3 468 021,25
PERCISTAND	Development of all thin-film PERovskite on CIS TANDem photovoltaics	850937	01/01/2020	30/06/2023	€ 4 997 437,50
GreEnergy	Wideband optical antennae for use in energy harvesting applications	101006963	01/01/2021	31/12/2024	€ 3 999 065,00
X-ROTOR	X-shaped Radical Offshore wind Turbine for Overall cost of energy Reduction	101007135	01/01/2021	31/12/2023	€ 3 900 008,75

## Data Availability

No underlying data are associated with this article.
