# Supplementary material for: Developing the next generation of renewable energy technologies: an overview of low-TRL EU-funded research projects
Source: Open Res Eur. 2023 Dec 28;3:8. Originally published 2023 Jan 11. [Version 2] doi: 10.12688/openreseurope.15276.2 (PMC10598454; doi:10.12688/openreseurope.15276.2)
Supplement: Peer review history

# Reviewer report: Reviewer response for version 1

**DOI:** 10.21956/openreseurope.16518.r35248

**Published:** 2023-10-24

**Authors:** Oskar Juszczyk

**Affiliations:** 1 University of Vaasa, Vaasa, Finland

Highly interesting report highlighting the importance of emerging technologies in the enhanced development of the renewable energy industry. Each project is roughly, but comprehensively described. This publication appears to be a conference wrap-up or report rather than research-based publication with scientific merits.

 More information about the background, context, and most importantly, aim and expected outcomes of the study is suggested.

 More relevant and up-to-date citations are highly recommended.

 Also, as TRL is implemented as a main conceptual tool, it should be thoroughly described, explaining each level to the reader. Are all the projects at 3-4 level? Perhaps it would be clearer to state the current level in the description of each project.

 More discussion is suggested in conclusions. What are the main results, outcomes of the study? How to successfully manage and monitor the development of the low-TRL renewable energy technologies? What can successful scenarios teach us? What are the practical and theoretical implications? 

 Please consider including these remarks, possibly too extensive for the Open Letter, but ensuring the overall quality improvement of the publication.

Where applicable, are recommendations and next steps explained clearly for others to follow? (Please consider whether others in the research community would be able to implement guidelines or recommendations and/or constructively engage in the debate)

Yes

Does the article adequately reference differing views and opinions?

Yes

Are all factual statements correct, and are statements and arguments made adequately supported by citations?

Partly

Is the rationale for the Open Letter provided in sufficient detail? (Please consider whether existing challenges in the field are outlined clearly and whether the purpose of the letter is explained)

Partly

Is the Open Letter written in accessible language? (Please consider whether all subject-specific terms, concepts and abbreviations are explained)

Yes

Reviewer Expertise:

Technology diffusion, renewable energy industry, innovation management

I confirm that I have read this submission and believe that I have an appropriate level of expertise to confirm that it is of an acceptable scientific standard, however I have significant reservations, as outlined above.

# Reviewer report: Reviewer response for version 1

**DOI:** 10.21956/openreseurope.16518.r35251

**Published:** 2023-10-24

**Authors:** Konstantinos Gkoumas

**Affiliations:** 1 European Research Executive Agency, Ispra, Italy

The paper provides an overview of 11 projects from an H2020 call. The paper reads well; however, it doesn’t seem to go beyond what is found in the CORDIS (Community Research and Development Information Service) database. Moreover, only ambitions of the projects and preliminary achievements or results are presented, since the projects are ongoing, and they were also assessed in an earlier stage.

1. What is missing in the paper to become academic is a section with a critical discussion from the authors to the technologies developed. The project specific sections are merely a positive presentation of projects and achievements from the point of view of the developers.
2. Ideally a table should be placed in an Annex with relevant project data, including acronym, title, cordis link or ID, total budget, start/end date. This would also facilitate the reader to inquire further on each project.
3. It would be good also to provide additional citations from project peer review papers from CORDIS results.
4. Check that all abbreviations are spelled out (e.g. COP - Coefficient of Performance?; CIGS - copper indium gallium diselenide). Then carbon dioxide (CO _2_) is spelled out (twice) in a later instance.

Where applicable, are recommendations and next steps explained clearly for others to follow? (Please consider whether others in the research community would be able to implement guidelines or recommendations and/or constructively engage in the debate)

Not applicable

Does the article adequately reference differing views and opinions?

Partly

Are all factual statements correct, and are statements and arguments made adequately supported by citations?

Yes

Is the rationale for the Open Letter provided in sufficient detail? (Please consider whether existing challenges in the field are outlined clearly and whether the purpose of the letter is explained)

Yes

Is the Open Letter written in accessible language? (Please consider whether all subject-specific terms, concepts and abbreviations are explained)

Partly

Reviewer Expertise:

Civil Engineering, Transport Engineering, Energy Harvesting, Knowledge Management

I confirm that I have read this submission and believe that I have an appropriate level of expertise to confirm that it is of an acceptable scientific standard, however I have significant reservations, as outlined above.

# Reviewer report: Reviewer response for version 1

**DOI:** 10.21956/openreseurope.16518.r34629

**Published:** 2023-10-17

**Authors:** Paul Adjei Kwakwa

**Affiliations:** 1 University of Energy and Natural Resources, Sunyani, Ghana

This work details key issues on eleven research and innovation projects under the same call of the EU's H2020 programme. It highlights the objectives of each project, current achievements where applicable and possible joint research activities are mentioned.

 Suggestions:

1. One suggestion is for the authors to indicate the duration for each project in the write up.
2. Also, if possible indicate the next phase of the project to be taken.
3. Since the success stories of the various projects have been captured, the authors should consider some challenges they encountered and how that was resolved, if any.

Where applicable, are recommendations and next steps explained clearly for others to follow? (Please consider whether others in the research community would be able to implement guidelines or recommendations and/or constructively engage in the debate)

Not applicable

Does the article adequately reference differing views and opinions?

Yes

Are all factual statements correct, and are statements and arguments made adequately supported by citations?

Yes

Is the rationale for the Open Letter provided in sufficient detail? (Please consider whether existing challenges in the field are outlined clearly and whether the purpose of the letter is explained)

Yes

Is the Open Letter written in accessible language? (Please consider whether all subject-specific terms, concepts and abbreviations are explained)

Yes

Reviewer Expertise:

Energy economics, environmental economics, development studies

I confirm that I have read this submission and believe that I have an appropriate level of expertise to confirm that it is of an acceptable scientific standard.

# Reviewer report: Reviewer response for version 1

**DOI:** 10.21956/openreseurope.16518.r31098

**Published:** 2023-05-16

**Authors:** Nikolaos E. Koltsaklis

**Affiliations:** 1 Faculty of Electrical Engineering, Czech Technical University in Prague, Prague, Czech Republic

I recommend the following modifications and additions:

1. Please mention clearly all the available techno-economic data of the new technologies.
2. Please make a comparison with similar technologies that already exist and highlight the expected advances.
3. Please add more citations to the technical description of each technology.
4. Please add a technology flowchart in each of the project sections.

Where applicable, are recommendations and next steps explained clearly for others to follow? (Please consider whether others in the research community would be able to implement guidelines or recommendations and/or constructively engage in the debate)

Yes

Does the article adequately reference differing views and opinions?

Yes

Are all factual statements correct, and are statements and arguments made adequately supported by citations?

Partly

Is the rationale for the Open Letter provided in sufficient detail? (Please consider whether existing challenges in the field are outlined clearly and whether the purpose of the letter is explained)

Partly

Is the Open Letter written in accessible language? (Please consider whether all subject-specific terms, concepts and abbreviations are explained)

Yes

Reviewer Expertise:

Energy systems

I confirm that I have read this submission and believe that I have an appropriate level of expertise to confirm that it is of an acceptable scientific standard, however I have significant reservations, as outlined above.
